# The Relationship Between Engagement and Neurophysiological Measures of Attention in Motion-Controlled Video Games: A Randomized Controlled Trial

**DOI:** 10.2196/games.5460

**Published:** 2016-04-21

**Authors:** Amber M Leiker, Matthew Miller, Lauren Brewer, Monica Nelson, Maria Siow, Keith Lohse

**Affiliations:** ^1^Auburn UniversityAuburn, ALUnited States; ^2^Rehabilitation Informatics LabSchool of KinesiolgyAuburn UniversityAuburn, ALUnited States

**Keywords:** games, engagement, motivation, eP3a, Kinect, exergame

## Abstract

**Background:**

Video games and virtual environments continue to be the subject of research in health sciences for their capacity to augment practice through user engagement. Creating game mechanics that increase user engagement may have indirect benefits on learning (ie, engaged learners are likely to practice more) and may also have direct benefits on learning (ie, for a fixed amount of practice, engaged learners show superior retention of information or skills).

**Objective:**

To manipulate engagement through the aesthetic features of a motion-controlled video game and measure engagement’s influence on learning.

**Methods:**

A group of 40 right-handed participants played the game under two different conditions (game condition or sterile condition). The mechanics of the game and the amount of practice were constant. During practice, event-related potentials (ERPs) to task-irrelevant probe tones were recorded during practice as an index of participants’ attentional reserve. Participants returned for retention and transfer testing one week later.

**Results:**

Although both groups improved in the task, there was no difference in the amount of learning between the game and sterile groups, countering previous research. A new finding was a statistically significant relationship between self-reported engagement and the amplitude of the early-P3a (eP3a) component of the ERP waveform, such that participants who reported higher levels of engagement showed a smaller eP3a (beta=−.08, *P*=.02).

**Conclusions:**

This finding provides physiological data showing that engagement elicits increased information processing (reducing attentional reserve), which yields new insight into engagement and its underlying neurophysiological properties. Future studies may objectively index engagement by quantifying ERPs (specifically the eP3a) to task-irrelevant probes.

## Introduction

Motion-controlled video game systems such as Microsoft Kinect, Playstation Move, and Nintendo Wii have seen a surfeit of research in recent years because these kinds of virtual environments provide the opportunity for motivating interactions and full-bodied movements and providing additional feedback beyond the body’s intrinsic sensory systems [ 1- 3]. One of the major areas of interest in this research is the potential for certain game mechanics to reliably increase participant engagement [ 4, 5]. Engagement has been defined as an affective quality or experience of a participant in a task that emerges from focused attention, aesthetic pleasures, perceptions of novelty, perceptions of usability, and the extent to which the participant feels involved in the task (ie, choices in the game have meaningful consequences [ 6, 7]). Viscerally pleasing stimuli, choice, clear mechanics/feedback, novelty/exploration, and adaptive difficulty are game mechanics thought to contribute to engagement [ 5, 8, 9]. Engagement is thus related to, but distinct from, motivation. Participants could be motivated to play a game, but if the game no longer offers adequate challenge, they may not be engaged by the game, potentially reducing future motivation.

Major reasons for using games in rehabilitation are the indirect effects that engagement might have on learning. That is, increased engagement might be beneficial for skill learning and rehabilitation because a participant will be inclined to practice more (ie, have greater compliance with therapy). Beyond this indirect benefit of engagement, recent evidence suggests that increased engagement during practice might also have a direct effect on learning [ 10]. In that study, Lohse et al manipulated the aesthetics of a gaming environment while keeping the amount of practice and the mechanics of the game constant. The group of participants who trained in the *game*group (complex, space-themed graphics with ambient and task-relevant sound) showed statistically superior retention and transfer performance compared to participants in the *sterile*group (simple, geometric graphics with no sounds), although the groups did not differ during practice (ie, gamification specifically enhanced learning). Furthermore, the game group self-reported statistically higher levels of engagement than the sterile group (using a language-adapted version of a user-engagement scale [ 6]). However, even though increased engagement was observed coincident to improved learning in the game group, individual engagement scores were not correlated with participant posttest performance, raising questions about the relationship between engagement and observed learning benefits.

This potential for engagement during practice to augment the learning of a novel motor skill was the impetus for the current experiment. Adapting the methods of Lohse et al [ 10], we conducted an electroencephalography (EEG) study in which participants practiced in either game or sterile conditions while task-irrelevant auditory probes were played at random intervals. Measuring event-related potentials (ERPs) in response to complex tones is a common research paradigm. In particular, we chose to focus on the amplitude of the early P3a (eP3a) component of the ERP waveform. The eP3a in response to auditory stimuli has been shown to be a reliable index of attentional reserve [ 11- 13]. That is, when more attention is being paid to the primary task (ie, more information being processed), the magnitude of the eP3a in response to an irrelevant tone will be lower as a consequence of fewer attentional resources being available to process the tone. Thus, we hypothesized that participants in the game-training group would show a reduced eP3a compared to the sterile group, suggesting that more attentional resources are absorbed by the task in the game condition than in the sterile condition. Consistent with the results of Lohse et al [ 10], we also hypothesized that the game group would show superior learning (ie, better performance on retention and transfer tests) compared to the sterile group. The experiment was powered specifically to detect these effects, but in order to follow up these a priori hypotheses, we also conducted exploratory analyses of the relationships between posttest performance, self-reported engagement, and eP3a amplitude.

## Methods

### Participants

A group of 40 right-handed participants was recruited through classes, flyers, and an online advertisement at Auburn University (17 male, 23 female). The average age of the participants was 22.6 (SD 3.15) years. Six participants indicated that they had used the Kinect system at some point in the past, but none of the participants had played in the last 3 months or regularly played before that (self-reported frequency 0 (SD 0.0) days/week). Many participants (n=31) indicated that they played some other form of motion-controlled game (mostly Nintendo Wii), with an average frequency of 0.2 (SD 0.61) days/week, and 36 participants indicated that they played games in some other medium (most commonly a mobile phone) with an average frequency of 1.34 (SD 1.59) days/week. Participants were randomly assigned to either the game group or the sterile group using blocked random assignment within sex to balance the groups. Participants self-reported no musculoskeletal or neurological impairments that would affect their performance, and all had normal or corrected-to-normal vision.

### Game Apparatus

**Figure 1:**
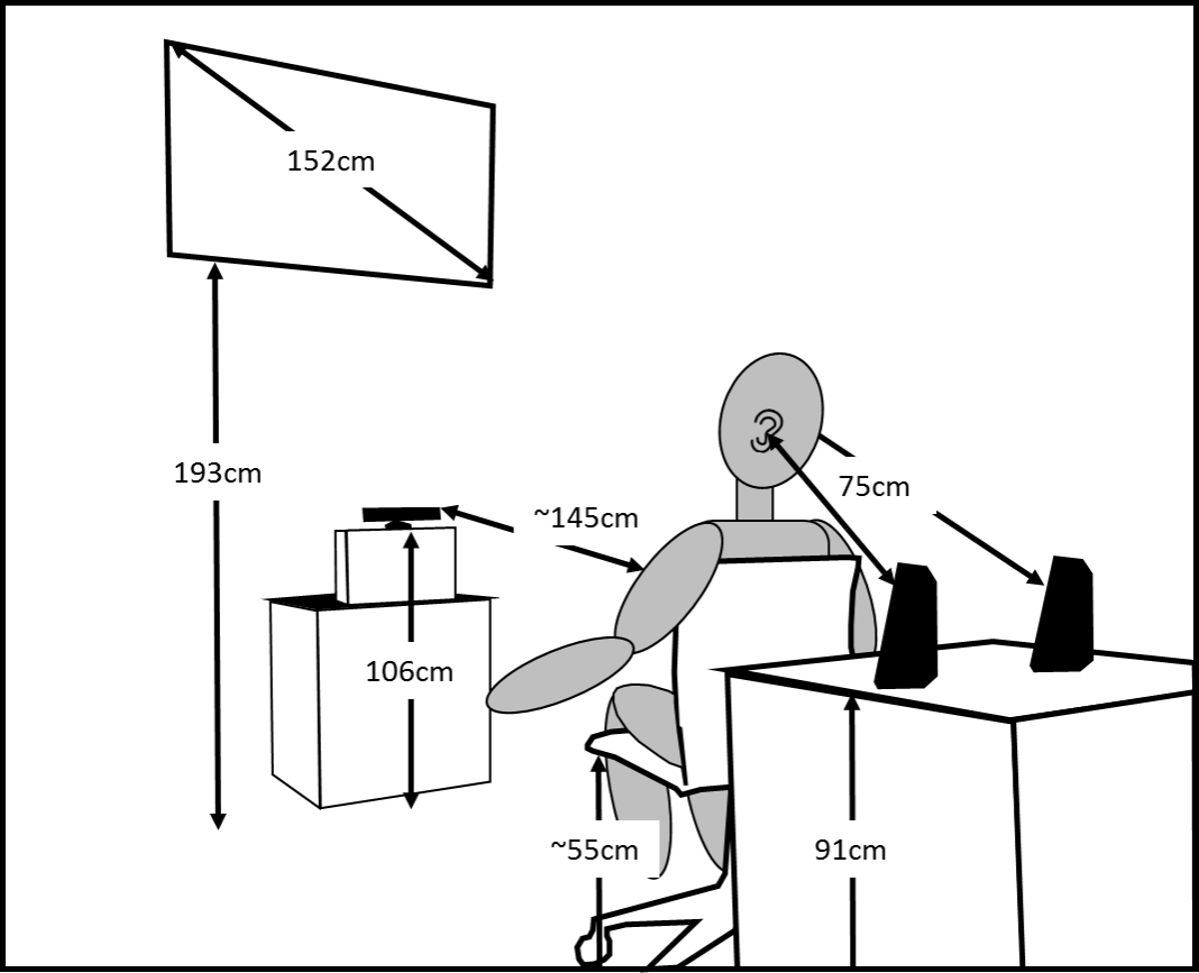
A schematic of experimental setup. The Kinect camera and speakers are shown in black.

Participants played a custom-built computer game written in Visual Studio 2013 using XNA Game Studio 4.0 and the Kinect SDK 1.8 using the Microsoft Kinect. The game was displayed on a 152 cm Samsung HDTV that was 193 cm above the ground (see Figure 1). The Kinect camera was placed 106 cm above the ground and approximately 145 cm away from the participant (who could move forward or back to improve tracking).

### Auditory Probes

Speakers for presenting the auditory probes were placed on a table 91 cm above the ground. The center of each speaker was set to an initial radial distance of 75 cm to the center of the ear of each participant. Probes consisted of 30 novel complex sounds (eg, door knock, dog bark, whistle) employed in previous studies using task-irrelevant auditory stimuli to index attentional reserve [ 11, 14, 15]. Probes were presented in random order at 75-95 dB SPL with interstimulus intervals varying randomly between 10 and 50 s.

### Procedures

All procedures were approved by the Internal Review Board of Auburn University (14-502 EP 1411). On day 1, participants provided written informed consent and completed an initial survey measuring handedness and past experience with video games. Next, participants were prepared for EEG recording while probe tones were played in order to habituate participants to the tones. Participants were told that these tones would be playing in the background during the experiment but they had nothing to do with the game.

**Figure 2:**
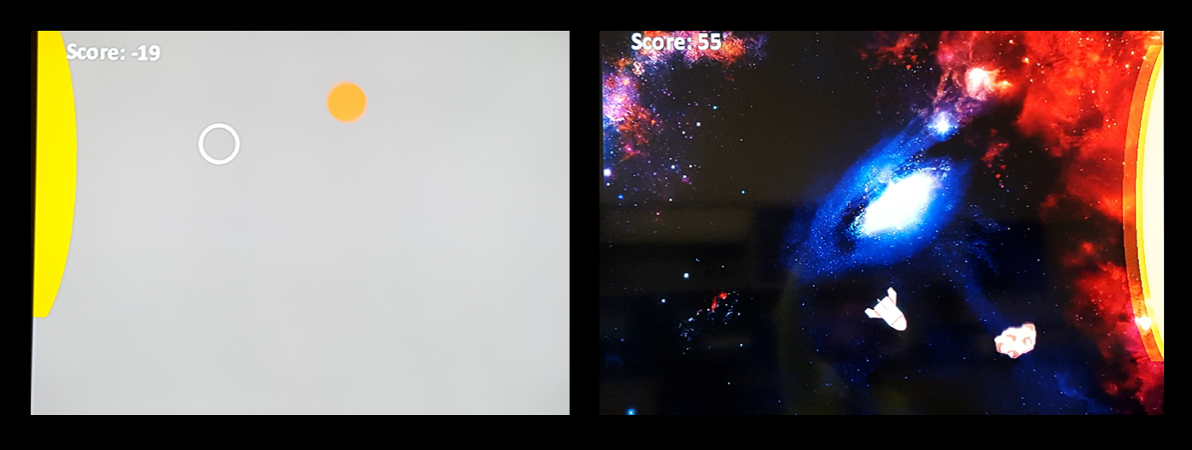
Sterile condition (left) and game condition (right).

The Kinect system was then calibrated to track the nondominant left hand, and all participants were given standardized instructions on how to play the game. In the game/sterile conditions, participants controlled the motion of a spaceship/cursor on the screen in order to catch asteroids/circles and throw them into yellow targets that would appear at the top, bottom, or sides of the screen. The two graphic types are shown in Figure 2; other than this difference, the game conditions were mechanically identical. All participants were instructed to catch the objects as quickly as possible and hit as many targets as they could. This combined speed-accuracy constraint was reinforced by participant in-game scores. Participants lost a single point for every 10 frames (approximately 167 ms) that they had not yet hit the target and scored 100 points for every target hit.

Following the standardized instructions, all participants completed a 20-trial pretest in both the same condition they would practice in and the opposite condition (40 trials total). The order of the pretest was counterbalanced across participants. The pretest was given under both test conditions to detect any potential baseline differences in the difficulty of the two conditions. No tones were played and no EEG data were recorded. Following the pretest, participants completed 200 practice trials in their given condition (game or sterile). EEG data were continuously recorded during this time period and probe tones were played. After 200 practice trials, participants were given the opportunity to rest. When ready, they began the second round of 200 practice trials in the same condition (400 practice trials total) with probe tones playing. Because the length of time required to complete the trials varied by how quickly participants caught and threw objects, the number of probe tones played varied as well (ie, faster players would hear fewer tones). The number of probe tones played ranged from 49 to 60 (mean 54.9, SD 2.86).

Approximately one week later (5-9 days with a median of 7 days), participants returned for retention (same condition as practice) and transfer (opposite condition) posttests. As with the pretest, the order of the posttests was counterbalanced across participants, but participants always completed the posttests in the same order they completed the pretest. Each posttest consisted of 20 trials (40 trials total). No tones were played, and no EEG data were collected during the posttest.

### Electroencephalograph Processing and Measures

Scalp EEG was collected from 20 channels of an EEG cap housing a 64-channel BrainVision actiCAP system (Brain Products GmbH) labeled in accord with an extended international 10-20 system [ 16]. EEG data were online-referenced to the left earlobe, and a common ground was employed at the FPz electrode site. Electrode impedances were maintained below 25 kΩ throughout the study, and a high-pass filter was set at 0.016 Hz with a sampling rate of 250 Hz. The EEG signal was amplified and digitized with a BrainAmp DC amplifier (Brain Products GmbH) linked to BrainVision Recorder software (Brain Products GmbH).

EEG data processing was conducted with BrainVision Analyzer 2.1 software (BrainProducts GmbH). Data were rereferenced to an averaged ears montage, band-pass filtered between 0.1 and 30 Hz with 24-dB rolloffs with a 60 Hz notch employing a zero phase shift Butterworth filter. Next, eye blinks were reduced employing the independent component analysis (ICA)-based ocular artifact rejection function within the BrainVision Analyzer software (electrode FP2 served as the vertical electrooculogram channel; BrainProducts, 2013). This function searches for an ocular artifact template in channel FP2 and then finds ICA-derived components that account for a user-specified (70%) amount of variance in the template-matched portion of the signal from FP2. These components were removed from the EEG signal, which was then reconstructed for further processing. ERPs were obtained by extracting the epoch of 200 ms prior to probe onset through 800 ms postprobe, then baseline-corrected with reference to the preprobe interval. Next, ERPs containing changes of more than 100 µV within a moving 200-ms window were excluded from subsequent analysis. This resulted in 1.3% of trials being rejected. The remaining trials were then averaged. Visual inspection of participants’ average waveforms revealed substantial interindividual differences in component latencies. Accordingly, the adaptive mean amplitude quantification technique was employed [ 17] to quantify the N1, eP3a, and late P3a (lP3a) components, although the primary component of interest was the eP3a. For each participant, a 40-ms time window was centered on the peaks within the 200-300 ms and 350-450 ms time ranges for the eP3a and lP3a components, respectively, and a 20-ms time window was centered on the peak within the 100-200 ms range for the N1 component. Mean amplitude was calculated for each component within the time window at the electrode where the component was maximal when averaged across all participants. This resulted in the N1 and eP3a being calculated at Cz and the lP3a being calculated at Fz.

### Survey Measures

Following the end of practice on day 1, participants completed a posttraining survey that included a language-adapted version of a user-engagement scale developed in the human-computer interaction literature [ 6] and a language-adapted version of the Intrinsic Motivation Inventory (IMI) [ 18] edited to include only the interest/enjoyment, perceived competence, effort, and pressure/tension subscales.

### Statistical Power and Analyses

All statistical analyses were conducted using SPSS version 22.0 (IBM Corp). The experiment was designed to test two a priori hypotheses: (a) the game group would show superior learning relative to the sterile group as measured by in-game performance on the retention and transfer tests and (b) the game group would show a decreased eP3a relative to the sterile group.

We operationally defined the learning effect as the interaction of test (pre- vs post-) and training condition (game vs sterile) in a mixed-factorial analysis of variance (ANOVA). Assuming an alpha of .05, Cohen's *f*of .25 (a medium effect), and a positive correlation between the pretest and posttest ( *r*=.50), a total sample size of N=40 was needed to achieve approximately 80% power. For the eP3a, this sample size would also give us approximately 80% power to detect a Cohen's *f*of .45 (a large effect), assuming alpha is .05, operationally defined as the main effect of group (game vs sterile) in an independent samples *t*test. Power calculations used G*Power 3.1 [ 19].

Points scored in-game were analyzed in blocks of five trials (maximum 500 points per block). Learning was measured by points per block using a mixed-factorial ANOVA with a between-subjects factor of training condition (game vs sterile) and within-subject factors of test (pre- vs post-) and testing condition (game vs sterile).

N1, eP3a, and lP3a mean amplitudes were assessed by separate 1-way ANOVAs with a between-subjects factor of group.

For the engagement scale, we first conducted a reliability analysis of the questions for each subscale. The minimum Cronbach's alpha was .78, which allowed us to collapse across questions. Similarly, among average subscale scores the Cronbach's alpha was .83, allowing us to collapse across subscales into a single engagement score. Between-group differences in composite engagement were measured using independent samples *t*tests. Reliability for the subscales of the IMI was also quite good with a minimum Cronbach's alpha of .83, but among the average subscale scores the Cronbach's alpha was −.08, preventing us from collapsing across subscales into a composite IMI score. Between-group differences in IMI subscales were measured using independent samples *t*tests.

## Results

### No Differences in Learning Between Groups

As shown in Table 1, participants in both groups improved from pretest to posttest, which was confirmed by the main effect of test, *F*_1,38_=37.92, *P*<.001, η _p_^2^=0.50. However, there was no main effect of training condition, *F*_1,38_<1, and no test by training condition interaction, *F*_1,38_<1. The main effect of testing condition was not significant, *F*_1,38_=3.28, *P*=.08, η _p_^2^=0.08, although participants scored fewer points per block on average during the game test than during the sterile test (350.55 [SD 46.54] vs 363.99 [SD 38.77], respectively). None of the other interactions was statistically significant, with the largest being the 3-way interaction of test, training condition, and testing condition, *F*_1,38_=1.47, *P*=.23, η _p_^2^=0.04.

**Table 1:** Means (SDs) for performance variables and electrophysiological variables as a function of group (game, n=19; sterile, n=21).

	Pretest score ^a^	Posttest score ^a^	N1 ^b^	eP3a ^b^	lp3a ^b^
Game, mean (SD)	330.18 (58.32)	385.27 (34.07)	−8.59 (5.76)	7.92 (3.90)	3.47 (3.82)
Sterile, mean (SD)	322.14 (56.61)	391.50 (37.79)	−9.99 (4.00)	10.30 (5.30)	3.18 (2.24)

^a^Pretest and posttest scores are in points per block (maximum of 500) and refer to average performance across the two different test-types.

^b^N1, eP3a and lP3a are in µV.

### No Differences in Engagement Between Groups

As shown in Table 2, there was no difference between groups on the engagement scale overall, *t*_38_=−0.72, *P*=.48. Similarly, there were no significant differences in the subscales of focused attention, endurability, novelty, or perceived involvement. The difference in the usability subscale was not statistically significant ( *t*_38_=−1.86, *P*=.07), and although the difference in the aesthetics subscale was statistically significant ( *t*_38_=−2.07, *P*=.04), neither of these differences was significant after correcting for multiple comparisons (Bonferroni correction). On the IMI subscales, Table 3, the only statistically significant difference was in competence ( *t*_38_=−2.45, *P*=.02), but this difference was not significant following a correction for multiple comparisons (Bonferroni correction).

**Table 2:** Means (SDs) for the overall engagement scale and subscales as a function of group (game, n=19; sterile, n=21). The maximum on the engagement and IMI subscales is 7.

Engagement	Overall	FA ^a^	US ^b^	AS ^c^	EN ^d^	NO ^e^	IN ^f^
Game, mean (SD)	4.08 (1.03)	4.32 (1.40)	5.13 (1.23)	3.99 (1.46)	3.96 (1.18)	3.05 (1.61)	4.04 (1.45)
Sterile, mean (SD)	3.87 (0.84)	4.16 (1.02)	4.49 (0.96)	3.12 (1.19)	3.67 (1.13)	3.30 (1.25)	4.48 (1.23)

^a^FA: focused attention.

^b^US: usability.

^c^AS: aesthetics.

^d^EN: endurability.

^e^NO: novelty.

^f^IN: perceived involvement.

**Table 3:** Means (SDs) for the subscales of the IMI as a function of group (game, n=19; sterile, n=21). The maximum on the engagement and IMI subscales is 7.

IMI subscales	I/E ^a^	CM ^b^	EF ^c^	P/T ^d^
Game, mean (SD)	3.68 (1.61)	5.30 (0.96)	4.98 (1.58)	5.18 (1.21)
Sterile, mean (SD)	4.02 (1.19)	4.52 (1.04)	5.37 (1.24)	4.74 (1.35)

^a^I/E: interest/enjoyment.

^b^CM: competence.

^c^EF: effort.

^d^P/T: pressure/tension (reverse-coded so higher numbers mean less pressure).

### A Trend for a Difference Between Groups in the eP3a

**Figure 3:**
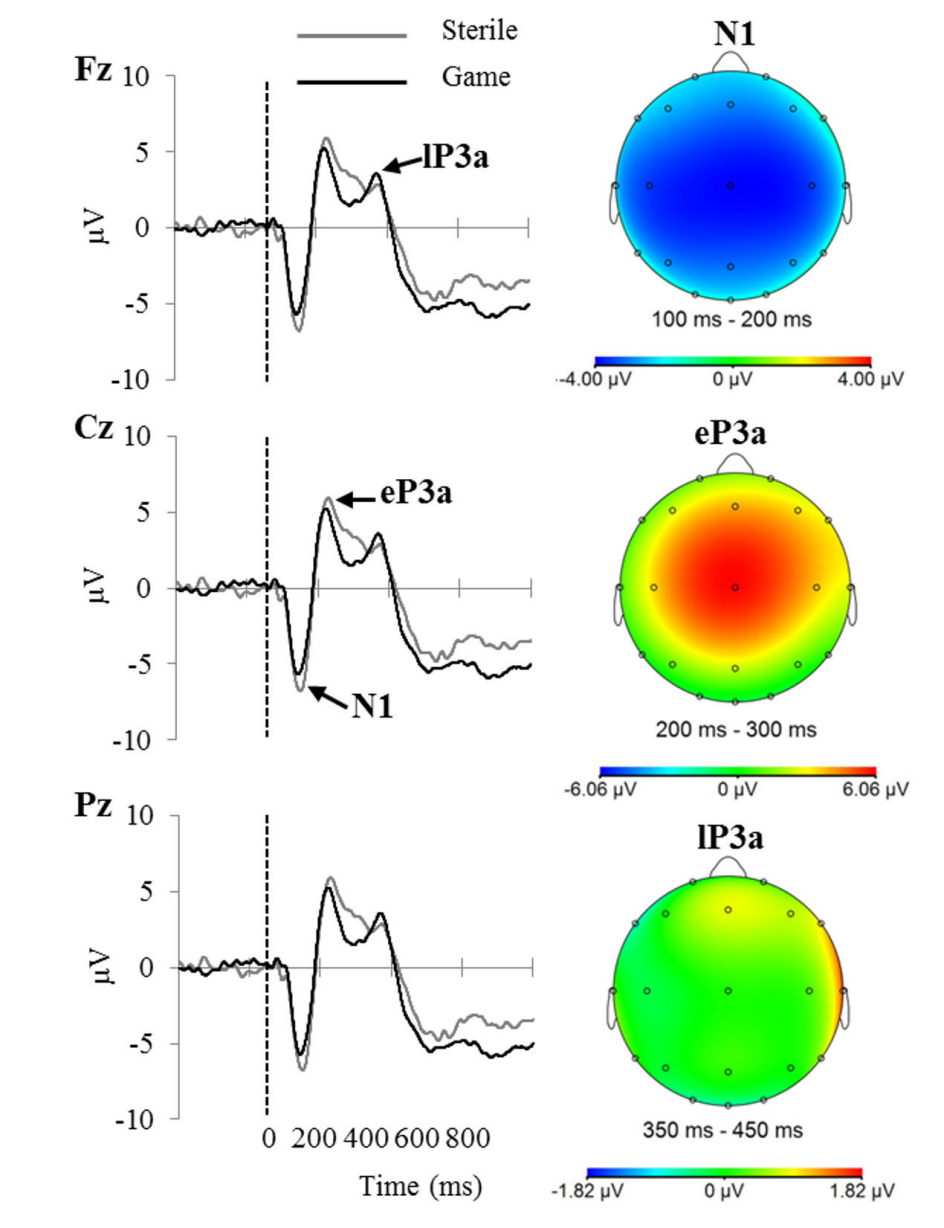
Grand average ERPs for the sterile and game groups (left). Topographies of the N1, eP3a, and lP3a components collapsed across groups (right).

The left panel of Figure 3displays grand average ERPs for the game and sterile groups at Fz, Cz, and Pz electrodes, and the N1, eP3a, and lP3a components are indicated. The topographies of the components collapsed across groups are displayed in the right panel. For EEG measures, there was no statistically significant difference in the N1 ( *t*_37_=−0.88, *P*=.38), eP3a ( *t*_37_=1.60, *P*=.12), or lP3a ( *t*_37_=.29, *P*=.77). As shown in Table 1, however, the eP3a effect was in the predicted direction with a moderately large effect size (Cohen's *d*=.51).

### eP3a Is Correlated With Self-Reported Engagement, Controlling for Group

**Figure 4:**
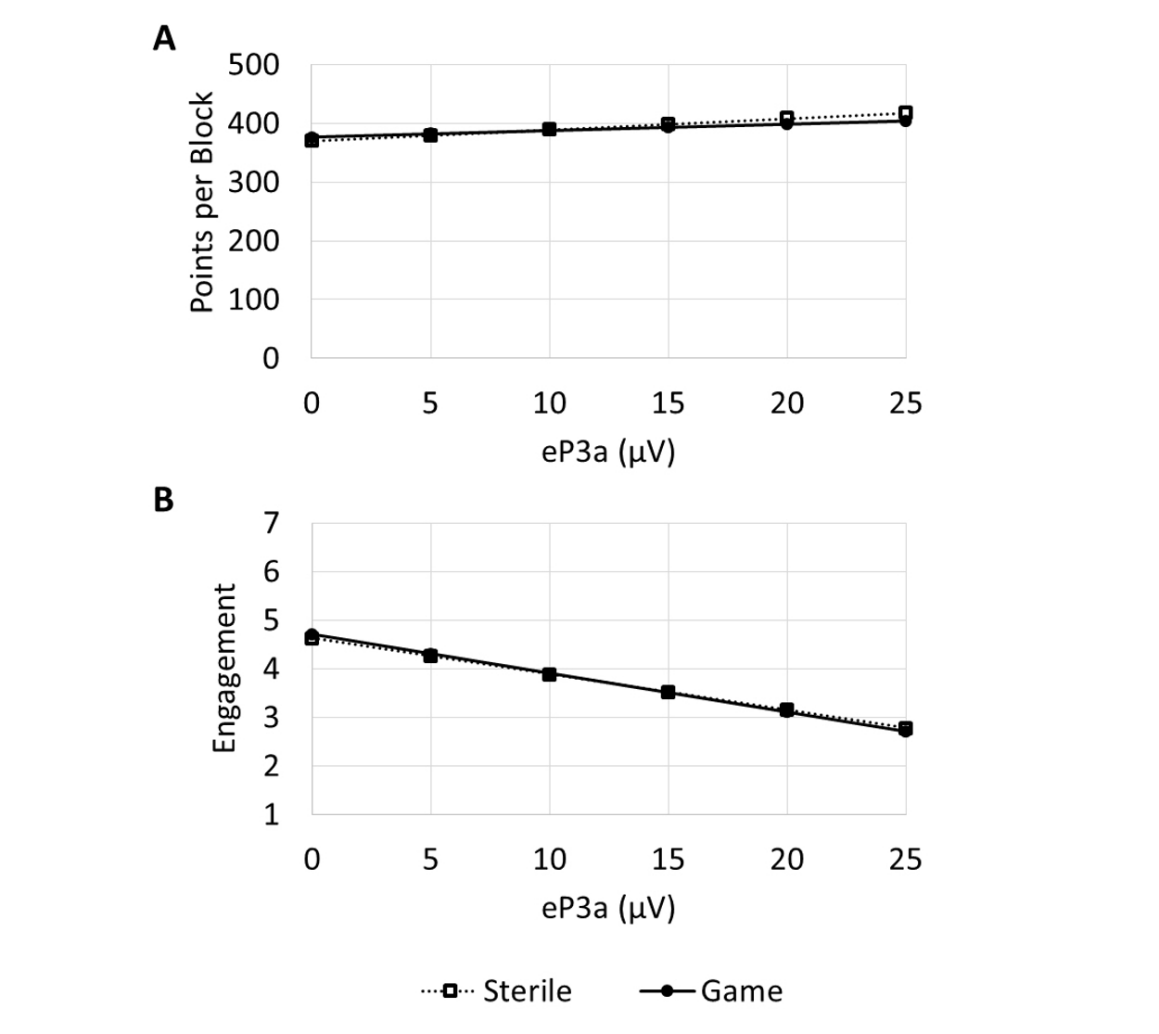
Predicted points per block on the posttest as a function of training condition and eP3a (top). Predicted engagement scores as a function of training condition and eP3a (bottom).

Although the a priori group difference in the eP3a was not supported, we were interested in exploring individual variability in the eP3a and how those differences related to engagement (overall engagement score) and learning (defined as posttest performance, given equivalent baselines). For these exploratory analyses, a step-up series of regression models was tested in which training condition, eP3a, and their interaction were regressed onto performance on the posttest (in points per block) and overall engagement scale scores (on a 1- to 7-point scale). Results of best fitting regressions for each series are shown in Multimedia Appendix 1. The predictions of the regression equations are shown in Figure 4. There were no reliable relationships observed between the eP3a and posttest performance even when controlling for training condition and the interaction. There was, however, a statistically significant negative relationship between the eP3a and self-reported levels of engagement. There was no evidence that this negative relationship changed as a function of group because the training condition by eP3a interaction was not significant. (Note that the relationship between eP3a and engagement was statistically significant even without controlling for group. However, given the difference between groups in the eP3a, the most appropriate analysis is the multivariable regression controlling for group.)

## Discussion

### Principal Findings

No significant learning or engagement effects were found between the game and sterile groups. Playing in an aesthetically pleasing game environment was not advantageous to learning; both groups showed similar levels of performance on the delayed retention and transfer tests. However, our data provide evidence that attentional reserve decreased proportional to self-reported engagement levels in both game environments. The significant negative relationship of the eP3a to self-reported levels of engagement adds to the theoretical understanding of engagement by providing physiological data showing that engagement elicits increased information processing. These new physiological data suggest increasing engagement is not simply a change in affective state but a change in cognitive processing as well.

Although the learning and engagement effects of Lohse et al [ 10] were not replicated, there are potential explanations for this lack of replication. First, the use of probe tones in the current experiment meant sounds could not be played in the game condition, which included both background music and action-specific sounds in the previous experiment. Second, in the current experiment participants were seated rather than standing to accommodate the EEG equipment. A limited movement space may have affected engagement during gameplay; participants in the previous experiment had greater freedom to move. A third possibility is that practicing 400 trials in one sitting, as opposed to 200 trials per day for two days in the previous experiment, may have led to boredom, especially if the participant wasn’t particularly challenged by the task.

Ultimately, this negative result complicates our previous conclusions about beneficial effects of engagement on learning. However, we cannot consider this study a failure to replicate the work of Lohse et al [ 10] because of the various experimental differences. The fact that neither the learning effect nor the engagement effect were found in this study may be reassuring, as there may still be a relationship between the two. Specifically, in the previous study gamification enhanced engagement and learning, whereas in the present study gamification failed to enhance engagement, possibly explaining why learning was not improved. However, the current null results do cast some doubt on the robustness of the previous effect, if not its validity. In order to validate the initial results, we are currently conducting a direct replication to see if the original learning and engagement effects can be obtained in a new sample of participants.

The negative relationship between engagement and the eP3a suggests that engagement causes a fundamental change in information processing and is not just an affective experience. The decreased amplitude of the eP3a in relation to higher self-reported levels of engagement indicates that more attentional resources are being used when players are more engaged in the game. In addition, this relationship was similar in both the game and sterile conditions. These physiological data give us a new insight to engagement. Although engagement is generally discussed with respect to affective consequences [ 4, 9], we have empirically demonstrated a neural correlate of cognitive resources being consumed with increased engagement. The eP3a in response to task-irrelevant tones is an objective, physiological correlate of engagement that could be used to measure engagement in many different populations across many different tasks. Future research may further examine the direct effect of engagement on learning through the eP3a.

Within the context of rehabilitation or other applications of serious games, the eP3a may provide a useful and relatively objective index of engagement. For instance, the eP3a could be a useful source of biofeedback allowing participants to “see” how much attention they have been allocating to their therapeutic tasks or a biomarker for adjusting difficulty, allowing the therapist to dynamically adjust the difficulty of practice to promote long-term learning (for a conceptually related study see Shirzad & Van der Loos [ 20]). Logistically, the constraints of collecting EEG data to measure engagement might make it a tool better suited for game designers; designers could play-test various game mechanics and measure corresponding changes in eP3a magnitude. As the benefits of using EEG systems increase (less expensive, less time-consuming, and more user friendly), there may be a place for EEG biofeedback in a routine clinical setting. At the moment, however, it is probably more feasible to measure motivation and engagement using validated survey measures. Although there are rehabilitation-specific measures of motivation [ 21], to our knowledge the only validated measures of engagement come from the human-computer interaction literature [ 6, 7]. Future research should adapt existing or develop new engagement scales specific to rehabilitation.

### Limitations

A limitation of the present study is that participants were not as engaged in either condition as they were in the previous experiment [ 10]. Overall engagement scores were lower, which may have been affected by the changes in the experimental paradigm to accommodate additional EEG measurement (even the simple act of wearing the EEG cap for a prolonged period could have negatively affected engagement). Although we cannot make strong conclusions comparing across experiments, it is likely that structural differences between the two practice conditions (eg, no sounds vs sounds, sitting vs standing, and limited range of motion vs freedom of movement) might explain why engagement scores were generally lower in the current experiment than in our previous research.

### Conclusion

Although there is some evidence that performing a complex motor skill in a stimulating game environment increases engagement and learning [ 10], the present study found no differences in engagement or learning between the game and sterile groups. While further research is needed to better understand the potential effects of engagement on learning, the current findings suggest we can predict individual differences in engagement with the event-related potential component, eP3a. Not only do these results hold theoretical importance because they give more information about the nature of engagement, the results can contribute to real-world solutions for health and rehabilitation research. The eP3a has the potential to become an objective measure of engagement in studies of games for rehabilitation patients adjusting to numerous disabilities.

## Multimedia Appendix 1

Details of regression models exploring the relationship between the eP3a, posttest performance, and self-reported engagement.

## Abbreviations

EEG: electroencephalography
eP3a: early P3a
ERP: event-related potential
ICA: independent component analysis
IMI: Intrinsic Motivation Inventory
lP3a: late P3a
