# Supplementary material for: The Relationship Between Engagement and Neurophysiological Measures of Attention in Motion-Controlled Video Games: A Randomized Controlled Trial
Source: JMIR Serious Games. 2016 Apr 21;4(1):e4. doi: 10.2196/games.5460 (PMC4858597; doi:10.2196/games.5460)
Supplement: Supplementary file 1 [file games_v4i1e4_app1.pdf]

Multimedia Appendix 1

Details of regression models exploring the relationship between the eP3a, post-test performance, and self-reported engagement.

Model 1: Post-Test Score = Training Condition + eP3a

	SS	Df	MS	*F*	R^2 Change
Regression	2361.43	2	1180.72	0.92	0.045
Residual	46104.61	36	1280.68		
Coefficients	β	SE	*t*-value	*p*-value	
Intercept	372.63	12.84	29.03	<0.001	
Condition	−0.32	5.93	−0.05	0.96	
eP3a	1.64	1.26	1.30	0.20	

Model 2: Engagement Score = Training Condition + eP3a

	SS	Df	MS	*F*	R^2 Change
Regression	5.09	2	2.54	3.20	0.14
Residual	28.75	36	0.79		
Coefficients	β	SE	*t*-value	*p*-value	
Intercept	4.67	0.32	14.57	<0.001	
Condition	0.01	0.15	0.09	0.93	
eP3a	−0.08	0.03	−2.42	0.02	

Note. Regression coefficients are not standardized and are thus interpretable in their natural units. For the Training Condition variable, Sterile = '−1' and Game = '1'. R^2 Change refers to the additional variance explained in the current model beyond a model with Training Condition only. The addition of the Training Condition X eP3a interaction did not improve either model.
